# Intraoperative Neurophysiological Monitoring During Adult Brainstem Glioma Resection: Association Between Electrophysiological Changes and Early Neurological Outcome

**DOI:** 10.3390/curroncol33080481

**Published:** 2026-08-15

**Authors:** Giada Pauletto, Benjamin Skrap, Barbara Tomasino, Christian Lettieri, Elisa Ecoretti, Miran Skrap, Andrea Landi, Luca Denaro, Marco Vindigni, Massimo Robiony, Mariarosaria Valente, Tamara Ius, Lorenzo Verriello

**Affiliations:** 1 Neurology Unit, Head-Neck and Neuroscience Department, Santa Maria della Misericordia University Hospital, 33100 Udine, Italy; giada.pauletto@asufc.sanita.fvg.it; 2 Neurosurgery Unit, Head-Neck and Neuroscience Department, Santa Maria della Misericordia University Hospital, 33100 Udine, Italy; benjamin.skrap@asufc.sanita.fvg.it (B.S.); marco.vindigni@asufc.sanita.fvg.it (M.V.); 3 Scientific Institute, IRCCS E. Medea, 33037 Pasian di Prato, Italy; barbara.tomasino@lanostrafamiglia.it; 4 Clinical Neurology Unit, Head-Neck and Neuroscience Department, Santa Maria della Misericordia University Hospital, 33100 Udine, Italy; christian.lettieri@asufc.sanita.fvg.it (C.L.); mariarosaria.valente@asufc.sanita.fvg.it (M.V.); 5 Neurophysiology Unit, Head-Neck and Neuroscience Department, Santa Maria della Misericordia University Hospital, 33100 Udine, Italy; elisa.ecoretti@asufc.sanita.fvg.it; 6 Independent Researcher, 34151 Trieste, Italy; miran.skrap@gmail.com; 7 Academic Neurosurgery, Department of Neurosurgery, University of Padova, 35128 Padova, Italy; andrea.landi@unipd.it (A.L.); luca.denaro@unipd.it (L.D.); tamara.ius@unipd.it (T.I.); 8 Clinical Unit of Maxillofacial Surgery, Head-Neck and Neuroscience Department, Santa Maria della Misericordia University Hospital, 33100 Udine, Italy; massimo.robiony@asufc.sanita.fvg.it; 9 Department of Medicine (DMED), University of Udine, 33100 Udine, Italy

**Keywords:** adult brainstem glioma, intraoperative neurophysiological monitoring, MEP stimulation threshold, neurological outcome, brainstem surgery

## Abstract

Intraoperative neurophysiological monitoring (IONM) is widely used during brainstem surgery to reduce the risk of neurological injury, although its prognostic value in adult brainstem glioma (BSG) surgery remains unclear. We retrospectively analyzed 22 consecutive adults who underwent surgery for BSGs with multimodal IONM between 2010 and 2023. Monitoring included somatosensory evoked potentials (SSEPs), motor evoked potentials (MEPs), brainstem auditory evoked potentials (BAEPs), corticobulbar MEPs (CoMEPs), and cranial nerves electromyography. The primary endpoint was the association between intraoperative neurophysiological changes and postoperative neurological functional status at hospital discharge, assessed with the modified McCormick Scale (mMCS). Secondary endpoints included postoperative complications and length of hospital stay. SSEP warning criteria were met in 10 patients (45.5%), while 10 patients (45.5%) experienced a ≥50% reduction in MEP amplitude in at least one monitored muscle. Despite these findings, neither SSEP nor MEP amplitude changes were significantly associated with postoperative neurological status. Transcranial MEP stimulation thresholds increased significantly during surgery (*p* = 0.007), and threshold elevation was associated with postoperative complications (Kendall’s τ = 0.498, *p* = 0.007). BAEP recordings remained stable throughout all procedures. Conventional amplitude-based IONM changes were not associated with early neurological outcome after adult BSG resection. In contrast, increases in MEP stimulation threshold may represent a sensitive indicator of postoperative complications. Further prospective studies are warranted.

## Simple Summary

Adult brainstem gliomas are rare tumors located in one of the most functionally critical regions of the central nervous system. Surgical treatment carries a substantial risk of neurological injury, making intraoperative neurophysiological monitoring (IONM) an important tool for preserving neural function. In this retrospective study, we evaluated the association between intraoperative electrophysiological changes and early postoperative neurological outcomes in 22 adults who underwent surgical resection of brainstem gliomas. Somatosensory and motor evoked potential changes were frequently observed during surgery; however, conventional amplitude-based warning criteria were not associated with neurological status at hospital discharge. In contrast, increases in motor stimulation thresholds were significantly associated with postoperative complications. These findings suggest that dynamic changes in motor threshold may provide clinically relevant information beyond traditional amplitude-based monitoring parameters. Larger prospective studies are needed to confirm the prognostic value of specific IONM metrics and to optimize intraoperative monitoring strategies during brainstem glioma surgery.

## 1. Introduction

Brainstem gliomas (BSGs) are uncommon neoplasms, accounting for approximately 1–2% of adult primary brain tumors and up to 20% of pediatric brain neoplasms [1,2]. They comprise a biologically, radiologically, and clinically heterogeneous group of tumors [3].

Prognosis and therapeutic management are strongly influenced by clinical presentation, radiological features, histopathological findings, and molecular profile [4,5].

Molecular profiling studies have demonstrated that adult BSGs exhibit distinct biological and molecular characteristics compared with both pediatric brainstem gliomas and adult supratentorial gliomas [3,4,6,7,8].

Despite significant advances in molecular characterization and neuroimaging, the optimal management strategy for adult BSGs remains controversial.

Historically, surgical management of BSGs was limited by the eloquent anatomy and critical functional role of the brainstem. Although resection was traditionally reserved for focal or exophytic lesions and diffuse intrinsic tumors were generally managed non-surgically, advances in microsurgical anatomy, identification of safe entry zones, diffusion tractography, intraoperative imaging, anesthetic techniques, and perioperative care have progressively expanded the role of surgery [6,7,9,10,11,12,13]. In carefully selected patients, surgery may achieve cytoreduction, relieve mass effect, and provide tissue for comprehensive histopathological and molecular characterization, thereby enabling personalized therapeutic strategies and more accurate prognostic stratification.

Among these advances, intraoperative neurophysiological monitoring (IONM) has emerged as a key adjunct, enabling real-time functional assessment of critical neural pathways and cranial nerve nuclei during brainstem surgery [14].

Previous studies on IONM in brainstem surgery have primarily focused on technical aspects, including cranial nerve mapping, floor-of-the-fourth-ventricle mapping, and optimization of monitoring strategies [15,16]. However, evidence regarding the prognostic significance of intraoperative neurophysiological changes remains limited, particularly in adult patients undergoing BSG resection [17,18]. Clarifying the predictive value of specific intraoperative neurophysiological changes may improve intraoperative decision-making, facilitate risk stratification, and enhance the interpretation of monitoring findings during BSGs surgery.

Therefore, the primary objective of this study was to evaluate the association between intraoperative neurophysiological changes and postoperative functional status in a consecutive cohort of adult patients undergoing surgical resection of BSGs. Secondary endpoints included postoperative surgery-related complications and length of hospital stay. Functional status was assessed using the modified McCormick Scale (mMCS) [19]. Although originally developed for patients with intramedullary spinal cord tumors, the mMCS is particularly suited to diseases affecting compact eloquent structures of the central nervous system, where small anatomical lesions may produce substantial neurological deficits despite relatively preserved global functional status. Because the brainstem shares these anatomical and functional characteristics with the spinal cord, we considered the mMCS more appropriate than broader disability scales, such as the modified Rankin Scale or the Karnofsky Performance Status, for assessing neurological functional changes in this patient population.

## 2. Materials and Methods

### 2.1. Study Design and Patient Selection

We conducted a retrospective single-center study including all consecutive adult patients with BSGs who underwent microsurgical resection at our Institution between January 2010 and December 2023.

Inclusion criteria were: (1) age ≥18 years; (2) radiological diagnosis consistent with brainstem glioma; (3) surgical treatment with IONM; and (4) availability of complete clinical data. Patients with a history of previous surgery, radiotherapy, or chemotherapy for the index lesion were excluded.

Clinical records, neuroimaging studies, operative reports including IONM data, histopathological findings, and follow-up information were systematically reviewed.

The following variables were collected from the medical records: demographic characteristics (age and sex), tumor-related variables (location, laterality, histological diagnosis and grade), presenting symptoms and neurological deficits, intraoperative neurophysiological findings, postoperative neurological outcome, surgical complications, and length of hospital stay. This study was reported in accordance with the Strengthening the Reporting of Observational Studies in Epidemiology (STROBE) guidelines.

The study was approved by the local Institutional Review Board (IRB) (reference number RIF. Prot IRB:185/2024 Tit III cl 13 fasc. 209/2024) and conducted in accordance with the Declaration of Helsinki and its subsequent amendments.

### 2.2. Clinical and Radiological Assessment

Clinical presentation at onset and neurological findings at admission were recorded. Neurological deficits were categorized into motor, sensory, and cranial nerve deficits, and cerebellar dysfunction.

Neurological status was assessed using the modified mMCS, both at hospital admission and discharge [19].

Preoperative magnetic resonance imaging (MRI) studies were reviewed for all patients. Tumors were classified according to the predominant brainstem compartment involved (midbrain, pons, or medulla oblongata), and tumor laterality was recorded.

Histopathological reports were reviewed to determine tumor subtype and grade according to the 2021 World Health Organization classification of tumors of the central nervous system [20].

### 2.3. Surgical Procedure

Surgical planning was based on tumor location, extent, and relationship to critical neurovascular structures. Patient positioning, surgical approach, and operative corridor were selected according to lesion anatomy and the surgeon’s judgment. Microsurgical resection was performed in all cases under multimodal IONM. Operative reports were reviewed to collect information regarding patient positioning, surgical approach, operative corridor, and surgeon-assessed extent of resection.

Extent of resection (EOR) was assessed by combining the surgeon’s intraoperative evaluation with postoperative MRI findings, including contrast-enhanced T1-weighted and T2/FLAIR sequences. EOR was categorized according to a modified EANS/EANO classification [21] as follows: gross-total/near-total resection (>90% tumor removal), subtotal resection (50–90% tumor removal), and partial resection or biopsy (<50% tumor removal).

### 2.4. Intraoperative Neurophysiological Monitoring

All surgical procedures were performed under multimodal IONM, following published international recommendations [16,22,23]. The monitoring protocol included somatosensory evoked potentials (SSEPs), motor evoked potentials (MEPs), brainstem auditory evoked potentials (BAEPs), corticobulbar motor evoked potentials (CoMEPs), free-running electromyography (EMG), triggered EMG, and electroencephalography (EEG), according to tumor location and the surgical corridor employed.

Cranial nerves V–XII were monitored using spontaneous and/or triggered EMG whenever deemed at risk based on tumor location and surgical approach. Direct nerve stimulation was performed with a bipolar concentric probe (0.3–1 mA, 1 Hz).

Upper-limb SSEPs were elicited by monophasic cathodic stimulation of the median nerve at the wrist (pulse duration 0.2 ms) and recorded from scalp electrodes placed at C3′ and C4′ according to the International 10–20 System. Band-pass filters were set between 2 Hz and 3 kHz.

MEPs were obtained through transcranial electrical stimulation (TES) delivered via corkscrew electrodes positioned at C3 and C4. Stimulation consisted of a train of 4–5 monopolar anodal pulses (pulse width 75 μs, range 25–100 μs; interstimulus interval 2 ms) delivered using a constant-voltage technique. Recordings were obtained bilaterally from the *extensor digitorum communis* (ECD) and *first dorsal interosseous* (FDI) muscles. Band-pass filters were set between 30 Hz and 10 kHz.

Baseline SSEP and MEP recordings were acquired before dural opening. Neurophysiological warning criteria were predefined as a persistent (>2–3 consecutive recordings) reduction greater than 50% in N20 amplitude for SSEP and MEP amplitudes compared with baseline values. These warning criteria were adopted because they represent the most widely accepted thresholds in current international recommendations and facilitate comparison with previous studies regarding IONM [16,23,24]. In the event of signal deterioration, surgical manipulation was temporarily interrupted and potential reversible causes, including systemic physiological factors and local surgical conditions, were promptly investigated. If necessary, the surgical strategy was modified accordingly.

CoMEPs were elicited by placing scalp electrodes at C3–Cz and C4–Cz, with short trains of 2–3 stimuli (pulse duration 200 μs; interstimulus interval 1.7–2 ms), and recorded from selected cranial-nerve-innervated muscles according to lesion location, including the *lateral rectus*, masseter, *orbicularis oculi*, *orbicularis oris*, vocal cords, *trapezius*, and tongue.

BAEPs were recorded using alternating click stimulation delivered at 60 dB above hearing threshold from ear-tips; stimuli were presented at a rate of 11.33 Hz, with band-pass filters set between 150 and 1500 Hz, and at least 500 responses were averaged for each acquisition. Intraoperative EEG was recorded with a reduced montage adapted to the operative field, typically including O1–Pz, O2–Pz, and additional frontocentral or parieto-occipital derivations.

### 2.5. Anesthesiologic Management

All procedures were performed under general anesthesia using a total intravenous anesthesia (TIVA) protocol.

Anesthesia was maintained with continuous infusions of propofol (5–8 mg/kg/h) and remifentanil (0.04–0.08 μg/kg/min).

### 2.6. Outcome Assessment

The primary outcome was the association between intraoperative neurophysiological changes and postoperative neurological functional status at hospital discharge, assessed using the mMCS [19].

Secondary outcomes were: postoperative complications, including neurological and non-neurological adverse events, and length of hospital stay.

### 2.7. Statistical Analysis

All statistical analyses were performed using IBM SPSS Statistics (Version 29.0).

Continuous variables were summarized as mean ± standard deviation (SD), whereas categorical variables were reported as frequencies and percentages. Given the relatively small sample size (*n* = 22) and the ordinal or non-normally distributed nature of several variables, non-parametric methods were employed.

Changes in neurological deficits between symptom onset and early neurological outcome were assessed using the McNemar’s exact test.

Individual neurological domains (motor, sensory, oculomotor, and other deficits), coded as binary variables (present/absent), were analyzed using McNemar’s test.

Preoperative and postoperative McCormick grades were compared using the Wilcoxon signed-rank test. Swallowing disturbances and PEG requirement were compared using the McNemar’s exact test.

Intraoperative neurophysiological variables were analyzed through paired comparisons between baseline (b) and final (f) amplitude of N20 and amplitude of upper limb MEPs using the Wilcoxon signed-rank test. Changes in the MEP stimulation threshold were quantified as the difference between the final and baseline stimulation threshold (Δ threshold), which was compared against zero using a one-sample *t*-test.

Associations between clinical variables and outcomes were evaluated using non-parametric approaches like Spearman’s rho for ordinal or continuous variables, and Pearson’s correlation coefficient for continuous variables. Associations between binary variables and nominal variables were assessed using Cramer’s V based on the χ^2^ test.

To explore whether EOR and tumor grade could act as potential confounding variables, their associations with postoperative complications and changes in MEP stimulation threshold were assessed using Spearman’s rank correlation coefficient.

All statistical tests were two-sided, and *p* values < 0.05 were considered statistically significant.

## 3. Results

### 3.1. Patient, Tumor, and Surgical Characteristics

Twenty-two patients were included in the analysis, including nine males (40.9%) and 13 females (59.1%), with a mean age of 35.6 ± 22.1 years.

Tumors were located on the left side in 45.5% of cases, on the right side in 36.4%, and were midline in 18.2%. The predominant anatomical location was the midbrain (68.2%), followed by the pons (22.7%) and medulla oblongata (9.1%).

Nineteen patients (86.4%) presented with at least one neurological deficit at clinical onset, whereas three patients (13.6%) presented exclusively with symptoms of intracranial hypertension (headache, nausea, and vomiting) without objective neurological deficits.

Histopathological examination revealed nine low-grade diffuse astrocytomas (40.9%), five pilocytic astrocytomas (22.7%), four high-grade diffuse astrocytomas (18.2%), three gangliogliomas (13.6%), and one ependymoma (4.5%).

Regarding the extent of resection, 11 patients (50.0%) underwent gross-total/near-total resection, seven (31.8%) underwent subtotal resection, and four (18.2%) underwent biopsy (Table 1).

No significant association was found between baseline neurological deficits and tumor histology (χ^2^ = 8.909, *p* = 0.259) or anatomical location (χ^2^ = 0.489, *p* = 0.783).

### 3.2. Clinical Outcome and Postoperative Complications

Neurological outcome was assessed at hospital discharge. Mean mMCS worsened slightly from 1.55 ± 0.60 preoperatively to 1.77 ± 0.87 postoperatively; however, no significant differences were observed between preoperative and postoperative scores (Z = −1.098, *p* = 0.272). Likewise, swallowing disturbances (*p* = 0.414) and the need for PEG placement (*p* = 0.317) did not significantly change after surgery.

No significant correlation was observed between baseline neurological deficits and postoperative mMCS score (*ρs* = 0.224, *p* = 0.321), nor between specific neurological deficits and postoperative mMCS (all *p* > 0.05).

Postoperative complications occurred in six patients (27.3%), including four cerebrospinal fluid fistulas and two cases of hydrocephalus. Complications were not significantly associated with tumor histology (χ^2^ = 9.186, *p* = 0.240), tumor location (χ^2^ = 0.657, *p* = 0.720), or preoperative neurological deficits (*ρs* = 0.243, *p* = 0.275). Furthermore, Spearman’s rank correlation analysis demonstrated no significant association between postoperative complications and either EOR (*ρs* = 0.243, *p* = 0.275) or tumor grade (*ρs* = 0.214, *p* = 0.339).

Mean length of hospital stay was 15.8 ± 7.4 days (range 8–35 days). The presence of neurological deficits at presentation was significantly associated with longer hospitalization (*ρs* = 0.516, *p* = 0.014). Among individual deficits, only oculomotor impairment was significantly associated with length of stay (*ρs* = 0.718, *p* < 0.001), whereas no significant associations were observed for other neurological deficits (all *p* > 0.05).

Length of stay was also significantly associated with the occurrence of additional clinical events during hospitalization (*ρs* = 0.496, *p* = 0.019), whereas only a trend toward an association with postoperative complications was observed (*ρs* = 0.422, *p* = 0.051).

### 3.3. Intraoperative Neurophysiological Monitoring and Clinical Outcome

All surgical procedures were performed under multimodal IONM.

Throughout the study cohort, BAEP recordings remained stable during surgery, with no persistent amplitude reductions, latency prolongations, or signal losses meeting predefined warning criteria. Consequently, no BAEP-related intraoperative alerts were generated, and BAEP data were not included in subsequent analyses.

CoMEP recordings were not consistently obtainable across all patients because of anatomical and technical constraints inherent to brainstem surgery. Given the incomplete availability of these data, CoMEP findings were excluded from statistical analyses. Nevertheless, no major CoMEP-related neurophysiological events requiring modification of the surgical strategy were observed.

**Figure 1:**
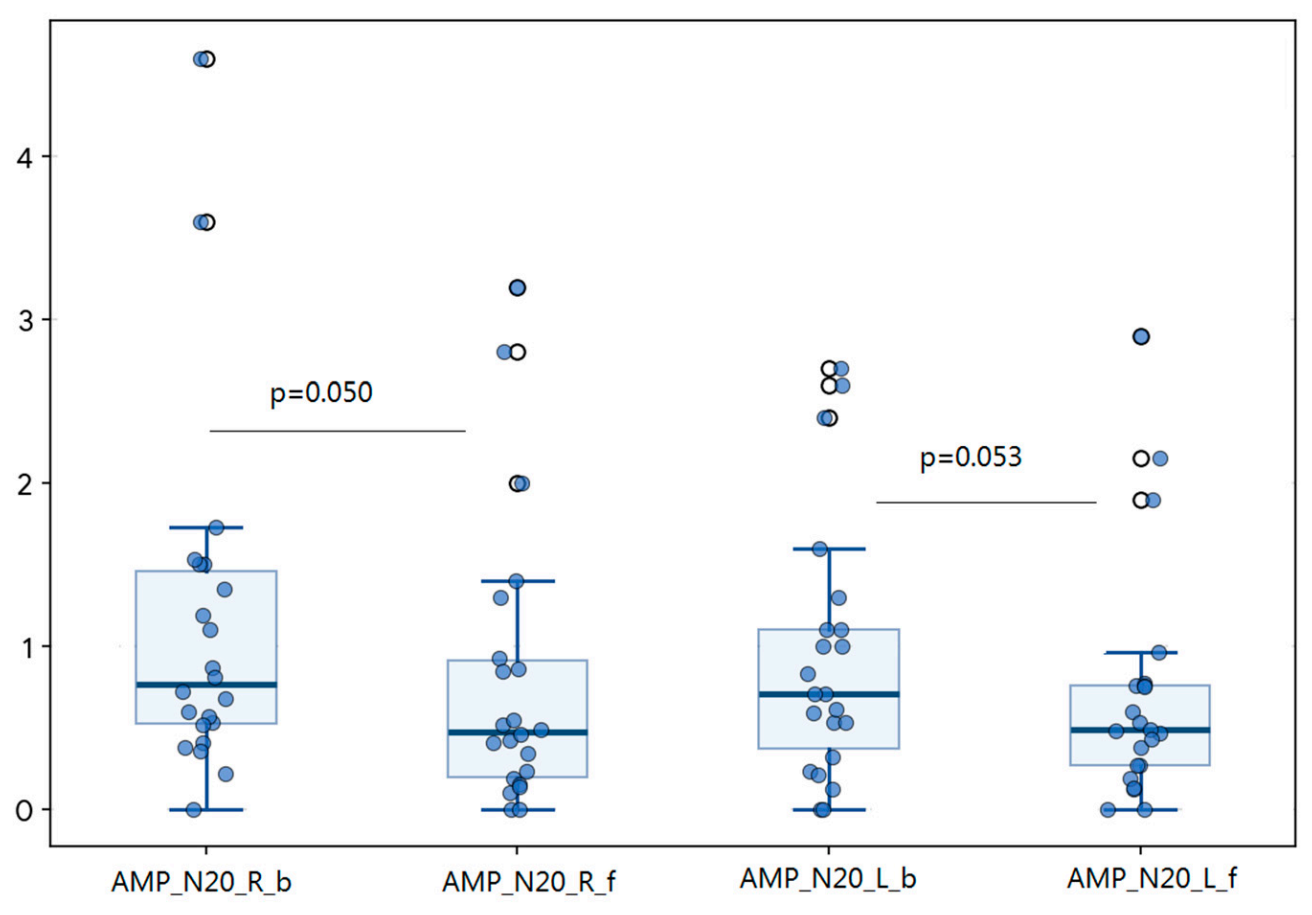
Intraoperative changes in somatosensory evoked potentials. Box plots showing changes in N20 amplitudes between baseline (b) and final (f) intraoperative recordings and individual data points. Right-sided N20 amplitude (AMP_N20_R) at baseline and at the end of surgery. Left-sided N20 amplitude (AMP_N20_L) at baseline and final recording. Both hemispheres demonstrated a trend toward reduced N20 amplitudes during surgery. The corresponding *p*-values are reported. Abbreviations: N20, cortical somatosensory evoked potential component; b, baseline; f, final recording.

SSEP changes fulfilling the predefined warning criterion were identified in 10 patients (45.5%). Bilateral SSEP deterioration occurred in four patients, whereas unilateral changes were observed in six cases, involving the right side in four patients and the left side in two patients. Analysis of SSEP recordings showed a decrease in N20 amplitudes at the end of the procedure compared with baseline. A one-sample Wilcoxon signed-rank test was performed to assess whether the change in N20 amplitude (ΔN20 amplitude = final − baseline) differed from zero. The change did not reach statistical significance for either the right N20 component (Z = −1.932, N = 22, *p* = 0.053) or the left N20 component (Z = −1.964, N = 22, *p* = 0.050). The median ΔN20 was −0.245 (range, −1.54 to 1.30) for the right N20 and −0.23 (range, −1.94 to 0.96) for the left N20. The corresponding effect size was *r* = 0.419 for both comparisons (Figure 1).

A reduction in MEP amplitude of at least 50% from baseline was observed in five patients (22.7%) for the right ECD, two patients (9.1%) for the right FDI, six patients (27.3%) for the left ECD, and five patients (22.7%) for the left FDI. Overall, 10 of 22 patients (45.5%) experienced a ≥50% decrease in MEP amplitude in at least one monitored muscle during surgery. Despite these intraoperative fluctuations, comparison of MEP amplitudes recorded at baseline and at the end of the procedure revealed no statistically significant differences in any monitored muscle group (Wilcoxon signed-rank test: right ECD, *p* = 0.420; right FDI, *p* = 0.277; left ECD, *p* = 0.733; left FDI, *p* = 0.770), suggesting overall preservation of corticospinal tract functional integrity throughout the surgical procedure. MEP stimulation thresholds at the end of surgery were significantly higher compared to baseline (Wilcoxon signed-rank test: Z = −2.701, *p* = 0.007). Similarly, the analysis of Δ threshold stimulation (final minus baseline threshold) revealed a significant increase over time (Z = −2.701, *p* = 0.007). Moreover, a larger Δ threshold was significantly associated with the occurrence of postoperative complications (Kendall’s τ = 0.498, *p* = 0.007) (Table 2). Additional analyses showed no significant association between changes in MEP stimulation threshold and either EOR (*ρs* = −0.008, *p* = 0.971) or tumor grade (*ρs* = −0.009, *p* = 0.967). No significant correlations were found between intraoperative SSEP changes, expressed as the difference between final and baseline N20 amplitudes, and postoperative functional status assessed by the modified McCormick Scale. Specifically, neither right N20 amplitude (*ρs* = 0.023, *p* = 0.916), left N20 amplitude (*ρs* = 0.162, *p* = 0.481), right ΔN20 amplitude (*ρs* = 0.046, *p* = 0.840), nor left ΔN20 amplitude (*ρs* = 0.229, *p* = 0.304) showed a significant association with postoperative mMCS scores.

Similarly, no significant associations were observed between intraoperative MEP changes, expressed as the difference between final and baseline amplitudes, and postoperative mMCS scores (right ECD Δ amplitude: *ρs* = 0.140, *p* = 0.535; left ECD Δ amplitude: *ρs* = −0.222, *p* = 0.321; right FDI Δ amplitude: *ρs* = −0.221, *p* = 0.324; left FDI Δ amplitude: *ρs* = −0.117, *p* = 0.603). Likewise, no significant correlation was found between changes in MEP stimulation threshold (Δ threshold) and postoperative functional status assessed by the modified McCormick Scale (*ρs* = 0.113, *p* = 0.526).

## 4. Discussion

The present study evaluated the relationship between IONM findings and early postoperative neurological outcome in a consecutive cohort of adult patients undergoing surgical resection of brainstem gliomas. The principal findings can be summarized as follows: (1) multimodal IONM was feasible in all surgical procedures; (2) transient or persistent alterations of SSEP and MEP amplitudes were relatively frequent during surgery; and (3) conventional amplitude-based warning criteria were not significantly associated with early postoperative functional outcome. Lastly, we observed an association between changes in MEP stimulation threshold and postoperative complications; however, this observation should be considered exploratory and hypothesis-generating, given the limited sample size and the multiple statistical comparisons performed.

Brainstem glioma surgery remains one of the most challenging procedures in neurosurgical oncology because of the high density of eloquent neural pathways and cranial nerve nuclei within a confined anatomical region [6,7,25,26]. Although advances in microsurgical techniques, neuroimaging, tractography, and perioperative management have expanded surgical indications, the risk of neurological morbidity remains substantial. In this context, IONM has become an essential adjunct to maximize resection while preserving neurological function [16].

Previous studies investigating IONM during brainstem surgery have primarily focused on technical aspects of monitoring and cranial nerve mapping, whereas evidence regarding the prognostic significance of intraoperative electrophysiological changes remains limited, particularly in adult BSGs [15,16,17,18]. Our findings contribute to this field by suggesting that conventional amplitude-based warning criteria may not fully capture the complexity of functional changes occurring during brainstem tumor resection.

Nearly half of the patients experienced SSEP changes meeting predefined warning criteria, and a similar proportion showed a ≥50% reduction in MEP amplitude in at least one monitored muscle. Nevertheless, these alterations were not associated with a worsening of modified McCormick scores at discharge. This observation may have several explanations. First, many intraoperative neurophysiological changes are transient and reversible following interruption of surgical manipulation, irrigation, or optimization of systemic physiological parameters. Second, the intrinsic plasticity and redundancy of sensorimotor pathways may allow preservation of clinical function despite measurable electrophysiological fluctuations. Finally, the use of multimodal monitoring may facilitate timely corrective actions that prevent progression from electrophysiological deterioration to permanent neurological injury. This observation is consistent with the findings of Slotty et al. in a large and heterogenous cohort of infratentorial lesions: they described a wide variation in incidence of IONM alterations during surgery in the posterior fossa, according to lesion locations and histopathological types, and recommended the simultaneous use of both modalities based on lesion location [18].

Interestingly, although MEP amplitudes remained globally preserved at the end of surgery, stimulation intensity required to elicit reliable responses significantly increased during surgery. Moreover, the magnitude of stimulation-threshold increase was significantly associated with postoperative complications. Similar observations have been reported in other neurosurgical settings, where threshold elevation has been proposed as an early indicator of functional compromise preceding complete MEP amplitude loss [27]. Abboud et al. demonstrated that a 20–50% increase in transcranial motor stimulation thresholds during supratentorial glioma surgery was associated with postoperative motor deficits, supporting the hypothesis that threshold elevation may reflect progressive impairment of corticospinal tract function secondary to ischemia, mechanical distortion, or reduced axonal excitability [27]. In the present study, however, threshold increases were associated with postoperative complications rather than neurological worsening. This observation should be interpreted cautiously, since most complications were non-neurological in nature. Consequently, the observed association may be influenced by the overall complexity of the surgical procedure and intraoperative physiological stress rather than by direct injury to descending motor pathways. Additional analyses revealed non-significant associations between EOR or tumor grade and either postoperative complications or changes in MEP stimulation threshold.

The absence of significant correlations between neurophysiological changes and early functional outcome should be interpreted cautiously. Functional status was assessed at hospital discharge, a relatively early time point at which neurological deficits may still be evolving. Some postoperative deficits observed immediately after surgery may subsequently improve, whereas delayed neurological deterioration may become evident only during longer follow-up. Therefore, the prognostic value of intraoperative neurophysiological alterations may be underestimated when considering only short-term outcomes.

An additional finding of our study was the stability of BAEP recordings throughout all procedures. No BAEP-related warnings were observed, suggesting either effective preservation of auditory pathways or limited involvement of structures at risk within the present cohort. Likewise, CoMEP data could not be systematically analyzed because of incomplete availability and technical limitations. Although these modalities did not contribute substantially to the statistical analyses, they remain important components of comprehensive multimodal monitoring during brainstem surgery, particularly when lesions are located in proximity to cranial nerve nuclei and corticobulbar pathways [16,28].

From a clinical perspective, our results support the concept that interpretation of IONM findings during BSGs surgery should not rely exclusively on binary amplitude-based warning criteria. Rather, a comprehensive evaluation integrating MEP stimulation thresholds, SSEP trends, cranial nerve monitoring, surgical anatomy, and intraoperative events may provide a more accurate assessment of neurological risk. Such an approach is particularly relevant in brainstem surgery, where subtle electrophysiological changes may reflect functional stress without necessarily translating into permanent neurological deficits [29].

Several limitations should be acknowledged. First, the retrospective design introduces an inherent risk of selection and information bias. Second, the sample size was relatively small, reflecting the rarity of adult BSGs and limiting statistical power. Third, the cohort was heterogeneous with respect to tumor histology, anatomical location, and extent of resection, factors that may influence both neurophysiological findings and clinical outcomes. Fourth, postoperative assessment was restricted to early neurological outcome at discharge, precluding evaluation of long-term functional recovery. Finally, incomplete availability of CoMEP data limited the analysis of corticobulbar pathway monitoring.

Despite these limitations, this study represents one of the limited investigations specifically addressing the relationship between intraoperative neurophysiological changes and clinical outcome in adult patients undergoing brainstem glioma resection. Future multicenter prospective studies with larger cohorts and standardized monitoring protocols are warranted to validate these findings, define clinically meaningful threshold-based warning criteria, and clarify the prognostic role of multimodal IONM in this highly challenging surgical population.

## 5. Conclusions

Multimodal intraoperative neurophysiological monitoring represents a valuable tool for functional surveillance during resection of adult BSGs. In this series, conventional SSEP and MEP amplitude changes did not predict early postoperative neurological outcome, whereas increases in MEP stimulation thresholds were associated with postoperative complications. Although this association should be interpreted cautiously because it may reflect greater surgical complexity rather than direct corticospinal tract injury, our findings suggest that MEP threshold changes may provide complementary information beyond conventional amplitude-based criteria. Larger prospective studies are needed to confirm their clinical utility and to refine intraoperative warning strategies in adult brainstem glioma surgery.

## Figures and Tables

**Table 1:** Baseline demographic and tumor characteristics of the study cohort.

Variable	Value
Patients, *n*	22
Age, years (mean ± SD)	35.6 ± 22.1
Male sex, *n* (%)	9 (40.9)
Female sex, *n* (%)	13 (59.1)
Tumor laterality, *n* (%)	
Left-sided	10 (45.5)
Right-sided	8 (36.4)
Midline	4 (18.2)
Tumor location, *n* (%)	
Midbrain	15 (68.2)
Pons	5 (22.7)
Medulla oblongata	2 (9.1)
Histology, *n* (%)	
Low-grade diffuse astrocytoma	9 (40.9)
Pilocytic astrocytoma	5 (22.7)
High-grade diffuse astrocytoma	4 (18.2)
Ganglioglioma	3 (13.6)
Ependymoma	1 (4.5)

**Table 2:** Intraoperative neurophysiological monitoring findings and electrophysiological changes during surgery.

IONM Parameter	Available Cases *n* (%)	Warning Criterion Met *n* (%)	Baseline (B) vs. Final (F) Recording	Statistical Analysis
BAEPs	22 (100)	0 (0)	No persistent latency prolongation, amplitude reduction, or signal loss	Not analyzed
CoMEPs	11 (50.0)	NA	Data not consistently available across the cohort	Excluded from analysis
SSEPs (right N20 amplitude)	22 (100)	10 (45.5)	Mild reduction at F recording	Wilcoxon signed-rank test: Z = −1.932, *p* = 0.053
SSEPs (left N20 amplitude)	22 (100)	10 (45.5)	Mild reduction at F recording	Wilcoxon signed-rank test: Z = −1.964, *p* = 0.050
Δ Right N20 amplitude (F − B)	22 (100)	—	Negative amplitude variation	Wilcoxon signed-rank test: Z = −1.932, *p* = 0.053
Δ Left N20 amplitude (F − B)	22 (100)	—	Negative amplitude variation	Wilcoxon signed-rank test: Z = 1.964, *p* = 0.050
MEP amplitude reduction ≥50% in ≥1 muscle	22 (100)	10 (45.5)	Final amplitudes not significantly different from baseline	All comparisons *p* > 0.05
MEP stimulation threshold	22 (100)	—	Significant increase at F recording	Wilcoxon signed-rank test: Z = −2.701, *p* = 0.007
Δ MEP stimulation threshold (F − B)	22 (100)	—	Positive threshold variation	Wilcoxon signed-rank test: Z = −2.701, *p* = 0.007
Association between Δ MEP stimulation threshold and postoperative complications	22 (100)	—	Positive correlation	Kendall’s τ = 0.498, *p* = 0.007

Abbreviations: BAEPs, brainstem auditory evoked potentials; B, baseline recording; CoMEPs, corticobulbar motor evoked potentials; F, final recording; IONM, intraoperative neurophysiological monitoring; MEPs, motor evoked potentials; N20, cortical somatosensory evoked potential component; SSEPs, somatosensory evoked potentials; Δ, change between final and baseline recordings.

## Data Availability

The data presented in this study are available from the corresponding author upon reasonable and scientifically justified request.
